# Cost Utility of Intensive Home Treatment Compared With Acute Psychiatric Inpatient Admission

**DOI:** 10.1001/jamanetworkopen.2025.12465

**Published:** 2025-05-27

**Authors:** Tamara Waldmann, Andreas Bechdolf, Konstantinos Nikolaidis, Sebastian von Peter, Gerhard Längle, Peter Brieger, Jürgen Timm, Lasse Fischer, Svenja Raschmann, Julian Schwarz, Martin Holzke, Sandeep Rout, Constance Hirschmeier, Johannes Hamann, Uwe Herwig, Johanna Baumgardt, Stefan Weinmann, Reinhold Kilian

**Affiliations:** 1 Section of Health Economics and Health Services Research, Department of Psychiatry and Psychotherapy II, Ulm University at Bezirkskrankenhaus Günzburg, Günzburg, Germany; 2 Department of Psychiatry, Psychotherapy, and Psychosomatics incorporating FRITZ am Urban and soulspace, Vivantes Hospital am Urban and Vivantes Hospital im Friedrichshain, Berlin, Germany; 3 Department of Psychiatry and Psychotherapy, Campus Charité Mitte, Charité - Universitätsmedizin Berlin, corporate member of Freie Universität Berlin and Humboldt-Universität zu Berlin, Berlin, Germany; 4 German Center for Mental Health (DZPG), Berlin-Potsdam site, Germany; 5 Faculty of Health Sciences Brandenburg, Brandenburg Medical University Theodor Fontane, Neuruppin, Germany; 6 Department of Psychiatry and Psychotherapy, DZPG, Immanuel Hospital Rüdersdorf, Brandenburg Medical School Theodor Fontane, Rüdersdorf, Germany; 7 Center for Psychiatry South Württemberg, Department of Psychiatry and Psychotherapy Zwiefalten, Zwiefalten, Germany; 8 Clinic for Psychiatry and Psychosomatics of Reutlingen (PP.rt), Academic Teaching Hospital of the University of Tübingen, Reutlingen, Germany; 9 General Psychiatry and Psychotherapy Division, Department of Psychiatry and Psychotherapy, University Hospital Tübingen and Medical Faculty of the University of Tübingen, Tübingen, Germany; 10 kbo-Isar-Amper Hospital Munich Region, Academic Teaching Hospital of Ludwig-Maximilians-University Munich, Haar near Munich, Germany; 11 Competence Center for Clinical Studies Bremen, Biometrics Department, University of Bremen, Bremen, Germany; 12 Center for Psychiatry South Württemberg, Department of Psychiatry and Psychotherapy I, University of Ulm, Weissenau, Germany; 13 Department of Psychiatry, Psychotherapy, and Psychosomatics, Vivantes Neukölln Hospital, Berlin, Germany; 14 Mainkofen District Hospital, Deggendorf, Germany; 15 Reichenau Center for Psychiatry, Academic Teaching Hospital University of Konstanz, Reichenau, Germany; 16 Psychiatric University Hospital Zurich, Zurich, Switzerland; 17 Department of Psychiatry and Psychotherapy, University Hospital Tübingen, Tübingen, Germany; 18 Scientific Institute of the AOK (WIdO), Berlin, Germany; 19 University Psychiatric Clinics (UPK) Basel, Faculty of Medicine, University of Basel, Basel, Switzerland; 20 Department of Psychiatry, Psychotherapy, and Psychosomatics, MediClin Clinic at Lindenhöhe, Offenburg, Germany; 21 Center for Integrative Psychiatry, University Hospital Schleswig-Holstein, Lübeck, Germany

## Abstract

**Importance:**

Intensive home treatment (IHT) is regarded as a safe and effective alternative to psychiatric inpatient care during acute crises.

**Objective:**

To estimate the cost-effectiveness of implementing IHT in comparison with inpatient treatment for persons with severe mental illness in acute crisis in the German health care system.

**Design, Setting, and Participants:**

This health economic evaluation was performed as part of a quasiexperimental nonrandomized trial conducted at 10 sites in Germany from January 2021 to December 2022. It included 200 patients with severe mental illness treated by IHT for acute crisis matched by means of propensity scores with 200 patients with severe mental illness receiving inpatient treatment. Participants were observed for 12 months. All analyses were conducted from January 15 to October 30, 2024.

**Main Outcomes and Measures:**

Incremental cost utility ratios (ICURs) were calculated for 12 months from the societal perspective and from the payer perspective of German statutory health insurance. ICUR uncertainty was estimated by nonparametric bootstrapping and estimated cost-effectiveness acceptability curves for maximum willingness to pay (MWTP) thresholds of €25 000 and €50 000.

**Results:**

The mean (SD) age of all 400 eligible study participants was 45 (16) years, and 264 (66%) were female. Overall, 374 were considered in the analysis. The ICUR point estimates were €48 786.43 from the perspective of statutory health insurance and €38 433.81 from the societal perspective. Acceptability rates for IHT being a cost-effective alternative compared with inpatient treatment from the societal perspective were 67% at an MWTP threshold of €25 000 and 50% at an MWTP of €50 000 and were 60% at an MWTP of €25 000 and 44% at an MWTP of €50 000 from the perspective of the statutory health insurance.

**Conclusions and Relevance:**

This economic analysis found that IHT for persons with severe mental illness in acute crisis is expected to be cost-effective compared with inpatient treatment, with a slightly higher acceptability probability for statutory health insurance than for the economy as a whole. Due to the high stochastic uncertainty, the study results suggest that more research is needed to assess the economic efficiency of IHT more clearly.

## Key Points

**Question:**

Is implementation of intensive home treatment (IHT) cost-effective compared with inpatient treatment for persons with severe mental illness in acute crisis in the German health care system?

**Findings:**

This economic evaluation of 374 participants found a probability of 67% that implementing IHT for patients with severe mental illness in acute crisis is cost-effective from the perspective of the statutory health insurance; and a probability of 60% that it is cost-effective from the societal perspective.

**Meaning:**

These findings suggest that IHT for persons with severe mental illness in acute crisis is expected to be cost-effective at a slightly higher acceptability probability compared with inpatient treatment.

## Teaser

This economic evaluation compares the cost-effectiveness of implementing intensive home treatment with inpatient treatment for persons with severe mental illness in acute crisis in the German health care system.

## Introduction

During the last 2 decades, intensive home treatment (IHT) has become increasingly popular as an outpatient alternative to psychiatric inpatient treatment for patients in acute psychiatric crises.^1,2,3,4,5,6,7,8,9,10,11^ Advantages of IHT compared with inpatient admissions are expected by mental health experts from the clinical perspective as well as from the economic perspective. From the clinical perspective, the provision of acute psychiatric care in the patients’ living environment is regarded as less disruptive for their daily routines and their social context and as less shameful or stigmatizing for the patients and their families.^4,11,12,13,14,15,16^ From the economic perspective, IHT is expected to be less costly because no hospital buildings and other hospital-related infrastructure are needed.^17^ As a consequence, IHT is expected to result in better outcomes, particularly with regard to patients’ quality of life at lower costs than acute inpatient care, which in health economic terms would result in being cost-effective.^1,17^

However, to date, only a few studies have provided evidence for the cost-effectiveness of IHT in comparison with inpatient treatment.^18,19,20^ Boege et al^18^ found that children and adolescents with a diagnosis of a child and adolescent mental disorder in need for acute mental health care who received an IHT after an initial inpatient admission revealed better improvement of psychosocial functioning at lower direct costs compared with those who received inpatient treatment of the usual duration. Kilian et al^19^ found better improvement of psychopathological symptoms at lower direct costs in adult patients with acute mental disorders who received IHT compared with those who received the usual inpatient treatment. While previous cost-effectiveness analyses used disease-specific outcome measures only, Barakat et al^20^ conducted the first cost-utility analysis (CUA) of an IHT compared with usual inpatient treatment using the gain of quality-adjusted life-years (QALYs) as a generalized outcome. The results of their study indicated an incremental cost utility ratio (ICUR) of €48 003 (to convert to US dollars, multiply by 1.09) for the gain of 1 QALY from the societal perspective and an ICUR of −€22 759 from the Dutch health care system perspective.^20^ However, due to differences between health care systems, the results of the Dutch study are not directly transferrable to Germany.

Only recently, results of the AKtiV study (in German: Aufsuchende Krisenbehandlung mit Teambasierter und Integrativer Versorgung),^11^ a quasiexperimental trial conducted at 10 sites in Germany, showed that patients with severe mental illness who received IHT for acute crisis care had fewer admissions to inpatient treatment or IHT during a 12-month follow-up period compared with patients receiving inpatient treatment for acute crisis care. In the present study, we investigated the CUA of IHT compared with inpatient treatment in Germany from the perspective of statutory health insurance and from the societal perspective based on the AKtiV trial.^11^

## Methods

This economic evaluation was a CUA from the societal perspective and the perspective of statutory health insurance as part of the AktiV trial^11,17^ conducted between January 2021 and December 2022 to evaluate the implementation of IHT in the German mental health care system. The design of the study was approved by the ethics committee of the Brandenburg Medical School Theodor Fontane and by the ethics committees of the participating study sites. Written informed consent was obtained from all participants. We report the health economic results in accordance with the updated version of the Consolidated Health Economic Evaluation Reporting Standards (CHEERS) reporting guideline.^21^ We published a health economic analysis plan as part D of the study protocol.^17^

### Study Population

The AKtiV study included patients with a primary diagnosis of a mental disorder according to *International Statistical Classification of Diseases, Tenth Revision* codes F0 to F6 during an acute mental health crisis assessed by a psychiatrist as requiring inpatient treatment. Other inclusion criteria were the existence of social and living conditions to be suitable for visits of the IHT team, informed consent of all adults living in the household (in the IHT study arm), and no associated child welfare risk for minor household members. Exclusion criteria were acute suicidality, aggressiveness toward others, a diagnosis of intellectual impairment, any form of commitment order, and insufficient German language skills.

### Comparators

Patients in the intervention group received inpatient equivalent psychiatric home treatment, a special form of IHT in Germany regulated by an agreement between medical institutions and health insurance entities in Germany. To be reimbursed by statutory health insurance, IHT must be provided by multiprofessional outreach teams consisting of psychiatrists, nurses, and at least 1 other professional group, such as psychologists, social workers, or occupational therapists. Patients’ eligibility for IHT must be assessed by a psychiatrist who also prepares the treatment plan. The IHT team provides comprehensive psychiatric and somatic health care, including diagnostics, medication, psychotherapy, and psychosocial interventions. The team was responsible for the patient 24 hours a day, 7 days per week and ensured daily face-to-face contact. A minimum of 6 encounters with a team member and at least 1 encounter with a psychiatrist per week must be ensured.

For each patient included in the IHT group, 1 patient who received usual inpatient treatment was included on the basis of propensity score matching.^17^ Propensity scores were estimated as the conditional probability of receiving IHT compared with inpatient treatment in each of the participating hospitals, including the total number of days in psychiatric inpatient or IHT treatment in the study center in the last 2 years, main psychiatric diagnosis, age, and gender as control variables.

### Setting and Location

We conducted the study at 10 sites in 4 federal states in Germany.^17^ All study sites were psychiatric hospitals or departments that recently implemented IHT.

### Time Horizon and Discounting

According to the duration of the primary study, the health economic analysis had a time horizon of 12 months. Due to this short time horizon, we applied no discounting of costs and outcomes.

### Cost Assessment

We assessed health and psychosocial service use by means of the German version of the Client Sociodemographic and Service Receipt Inventory.^22,23^ We completed the inventory interviews at baseline and at 6- and 12-month follow-up visits. Since no general unit cost list for the German health care system exists, we obtained information on unit costs from different sources (eTable 1 in Supplement 1). We computed 6-month direct costs for each point of assessment by multiplying used service units with unit costs. For estimating indirect costs, we multiplied the number of sick leave days and the number of days in early retirement by the mean daily gross salary for people in full-time employment in the year 2021.

We calculated annual costs of illness from the societal perspective by summing the direct and indirect costs assessed at the 6-month and 12-month follow-up as covering the 12-month follow-up period. For the analysis from the payer perspective, we summed only costs covered by statutory health insurance. If 6-month or 12-month follow-up data were not available, we carried forward baseline data for the purpose of an intention-to-treat analysis.

### Outcome Assessment

We applied the EuroQol 5-dimension 5-level questionnaire^24,25^ results for the assessment of health states as the basis for the estimation of QALYs.^26,27^ We estimated total QALYs by means of the area under the curve method.^28^

### Statistical Analysis

We estimated differences for QALYs and for each cost category by means of linear regression models. For consideration of skewed distribution of cost data, we applied robust estimation of standard errors by means of nonparametric bootstrapping with 5000 replications.^29,30,31^

We estimated the ICUR from the perspective of the statutory health insurance (payer perspective) as the cost difference from the payer perspective divided by the QALY difference, and the ICUR from the societal perspective as the cost difference from the societal perspective divided by the QALY difference. For assessing the stochastic uncertainty of the ICUR, we applied nonparametric bootstrapping with 10 000 replications.^28^ For the interpretation of the ICUR, we estimated cost-effectiveness acceptability curves for maximum willingness to pay (MWTP) threshold ranges between €0 and €125 000. For assessing the economic value of further investigations in this field, we estimated the value of information per person (VOIP) from the societal perspective and from the perspective of statutory health insurance.^28^ In accordance with recent recommendations for national MWTP thresholds for Germany,^32,33^ we indicated acceptability rates for thresholds of €25 000 and €50 000.

All analyses were conducted from January 15 to October 30, 2024, with Stata 17 (StataCorp LLC). A 2-sided *P* < .05 was considered statistically significant.

## Results

**Figure 1.**
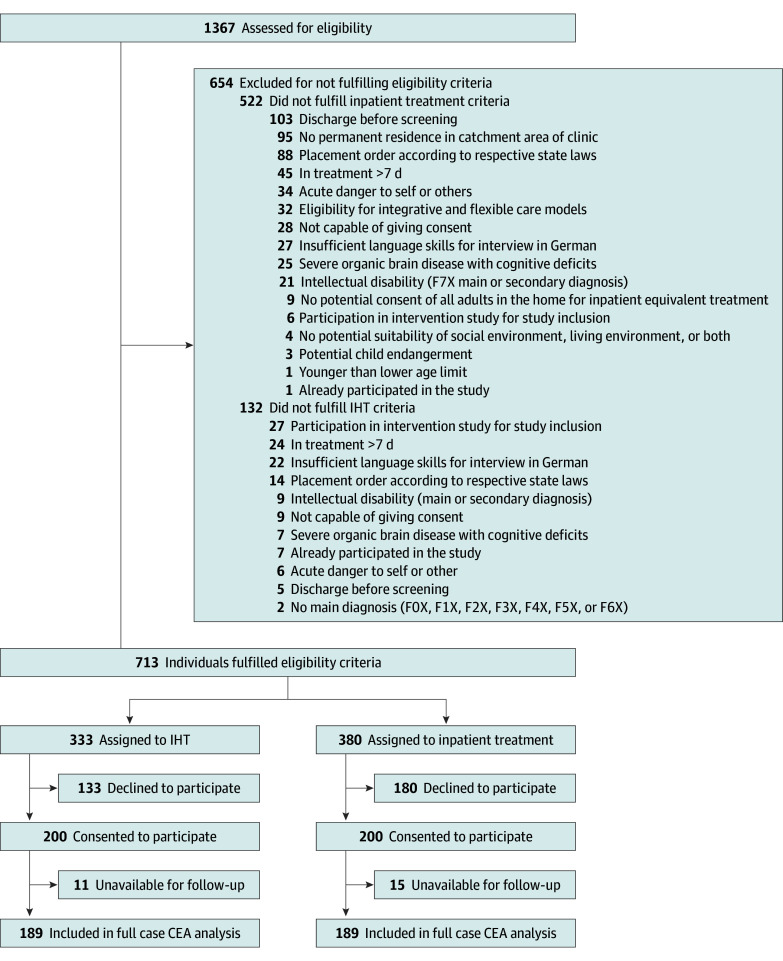
Study Flowchart CEA indicates cost-effectiveness acceptability; IHT, intensive home treatment.

Of 1367 patients assessed between January 2021 and December 2022, 713 fulfilled the eligibility criteria for IHT (Figure 1). Of 333 patients who were asked for IHT participation, 133 declined and 200 were included. Of 380 patients who were asked for inpatient treatment participation, 180 declined and 200 were included. In total, 11 participants from the IHT group and 15 participants from the inpatient treatment group were lost to follow-up. Overall, 189 participants in the IHT group and 185 participants in the inpatient treatment group were included in the full case analysis.

Among 400 study participants, the mean (SD) age was 45 (16) years; 264 (66%) were female and 136 (34%) were male; 54% of participants in the IHT group (107 of 200) and 64% in the inpatient treatment group (129 of 200) had no spouse; and 26% (51 of 200) in the inpatient treatment group and 32% (65 of 200) in the IHT group had a job. Most participants had a diagnosis from the spectrum of affective disorders, including major depression and bipolar disorder (n = 189 [47%]), followed by schizophrenia spectrum disorders (n = 86 [22%]) and other mental disorders (anxiety disorder, obsessive-compulsive disorder, somatoform disorder, or personality disorder) (n = 94 [24%]). During the last 2 years before study onset, participants in the IHT group had a mean (SD) of 1.6 (2.9) acute treatment episodes, while participants in the control group had 1.2 (2.3) episodes.

As indicated in the Table, participants in the IHT group incurred significantly lower annual costs for inpatient treatment (mean [SE] difference, −€9848.81 [€−1849.57]; *P* < .001) but significantly higher costs for IHT (mean [SE] difference, €8017.29 [€2475.24]; *P* < .001). Furthermore, participants in the IHT group incurred higher costs for outpatient medication (mean [SE] difference, €298.38 [€439.98]; *P* = .049) and for psychosocial outpatient care (mean [SE] difference, €811.72 [€854.57]; *P* = .02). However, sector-specific costs did not vary significantly with regard to direct, overall societal, or statutory health insurance costs.

**Table.** Annual Cost and Cost Differences by Cost Categories

Cost category	Annual cost by treatment, mean (SD), €^a^		Cost difference, IHT minus inpatient treatment^b^	
	Inpatient (n = 200)	IHT (n = 200)	Mean (SE), €	*P* value
Inpatient psychiatric	17 955.96 (19 995.38)	8107.15 (18 145.81)	−9848.81 (−1849.57)	<.001
Inpatient somatic	1871.57 (6479.86)	942.22 (3988.62)	−929.35 (−2491.24)	.68
Day hospital psychiatric	1557.05 (5567.45)	1649.20 (4986.85)	92.15 (−580.60)	.92
IHT	1520.83 (6216.04)	9538.12 (8691.28)	8017.29 (2475.24)	<.001
Outpatient psychiatric^c^	1286.21 (1488.23)	1607.54 (2105.96)	321.30 (617.73)	.20
Outpatient medication	952.02 (1297.79)	1250.40 (1737.77)	298.38 (439.98)	.049
Total costs covered by statutory health insurance	25 143.65 (23 674.16)	23 094.62 (21 023.98)	−2049.03 (−2238.83)	.36
Police or justice	378.51 (5352.94)	1.60 (22.63)	−376.91 (−5330.31)	.32
Outpatient psychosocial^d^	1207.49 (3094.21)	2019.21 (3948.78)	811.72 (854.57)	.02
Direct total cost	26 729.65 (24 310.80)	25 115.43 (21 358.07)	−1614.22 (−2952.73)	.28
Indirect cost	3048.25 (4839.77)	3220.30 (5005.83)	172.06 (483.73)	.72
Total societal cost	29 777.89 (25 148.06)	28 335.73 (23 155.28)	−1442.16 (−2399.55)	.55

Abbreviation: IHT intensive home treatment.

^a^ To derive approximate values in US $, multiply by 1.09.

^b^ Linear regression with robust standard errors based on nonparametric bootstrapping with 5000 replications.

^c^ Outpatient psychiatric includes psychiatrist, psychotherapist, outpatient clinic, family doctor, and specialized physician.

^d^ Outpatient psychosocial includes community mental health service, day center, community nurse, home assistant, community social worker, legal guardian, and phone counseling.

As indicated by the mixed-effects regression model, the EuroQol 5-dimension 5-level questionnaire utility values at the 3 follow-up assessments were slightly higher for participants in the inpatient treatment group than the IHT group but did not differ significantly (eTable 2 and eFigure 1 in Supplement 1). The regression model for the area under the curve–QALY estimator indicated no significant difference between study groups (b = −0.042; SE = 0.029; *P* = .17).

### CUA From the Perspective of Statutory Health Insurance

**Figure 2.**
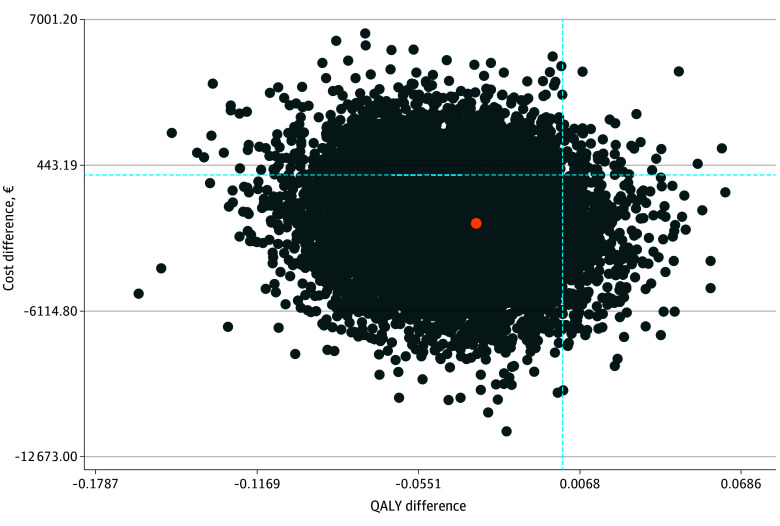
Incremental Cost Utility Ratio (ICUR) Variance From the Perspective of Statutory Health Insurance Variance in ICUR for the cost-effectiveness of implementing intensive home treatment vs inpatient treatment for people with severe mental illness in acute crisis in the German health care system from the perspective of statutory health insurance. The orange dot represents the point estimate of the ICUR; each dark blue dot, 1 of 10 000 ICURs estimated by nonparametric bootstrapping with 10 000 replications. QALY indicates quality-adjusted life-year.

The ICUR for the CUA from the perspective of statutory health insurance, calculated as the cost difference of −€2049.03 divided by the QALY difference of −0.042, equaled €48 786.43 and was located in the lower-left quadrant of the cost-effectiveness plane (Figure 2). The ICUR variance estimated by nonparametric bootstrapping with 10 000 replications shows the largest share of the variance (78%) in the lower-left quadrant, indicating a lower effectiveness at lower costs for the IHT intervention compared with inpatient treatment. This result was followed by 14% of the variance in the upper-left quadrant, indicating lower effectiveness at higher costs for the IHT compared with the inpatient treatment; 7% of the variance in the lower-right quadrant, indicating higher effectiveness at lower costs for the IHT compared with the inpatient treatment; and 1% of the variance in the upper-right quadrant, indicating higher effectiveness at higher costs for the IHT compared with inpatient treatment.

**Figure 3.**
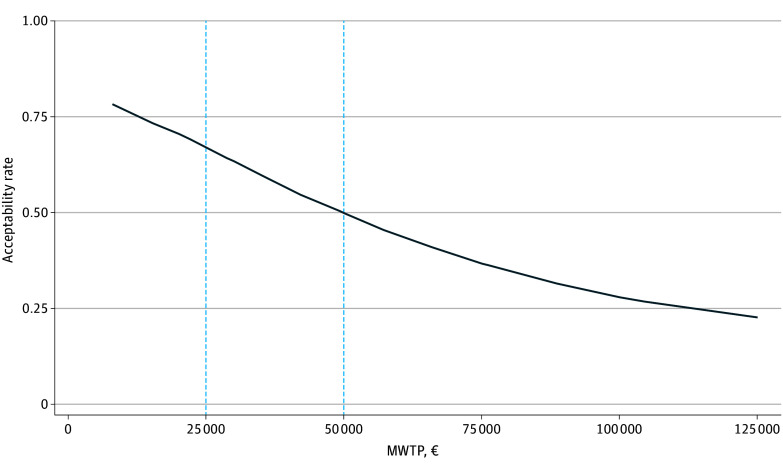
Cost-Effectiveness Acceptability Curve From the Perspective of Statutory Health Insurance Cost-effectiveness acceptability curve for the cost-effectiveness of implementing intensive home treatment vs inpatient treatment for people with severe mental illness in acute crisis in the German health care system from the perspective of statutory health insurance. The x-axis represents the range of maximum willingness to pay (MWTP) threshold values. The dashed vertical blue lines represent MWTP thresholds of €25 000 and €50 000 suggested for the German health care system. The y-axis represents the acceptability rates, indicating the probability that intensive home treatment would be accepted as cost-effective compared with inpatient treatment in the German health care system. The acceptability curve represents the change in the acceptability rate with increasing MWTP threshold.

The cost-effectiveness acceptability curve is shown in Figure 3. The results indicated that from the perspective of statutory health insurance, IHT compared with inpatient treatment would be accepted as cost-effective at an MWTP threshold of €25 000 with a probability of 67% and at an MWTP threshold of €50 000 with a probability of 50%.

**Figure 4.**
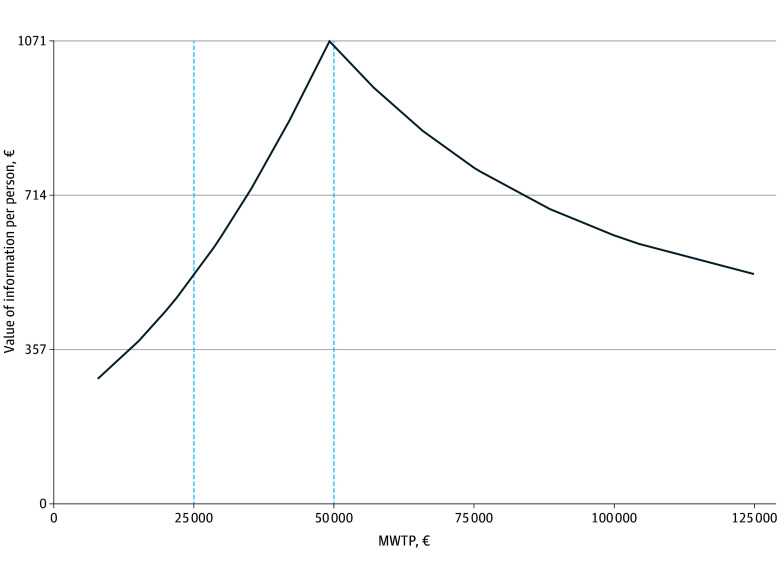
Value of Information Curve From the Perspective of Statutory Health Insurance The x-axis represents the range of maximum willingness to pay (MWTP) in threshold value. The dashed vertical blue lines represent the MWTP thresholds of €25 000 and €50 000 suggested for the German health care system. The y-axis represents the monetary value of acquiring perfect value of information per person (VOIP) in the population of potential service users. The curve represents the change in VOIP with increasing MWTP thresholds.

The results of the VOIP analysis are shown in Figure 4. The findings indicated a VOIP of €525 at an MWTP threshold of €25 000 increasing to €1062 at an MWTP threshold of €50 000.

### CUA From the Societal Perspective

The ICUR for the CUA from the societal perspective, calculated as the cost difference of −€1614.22 divided by the QALY difference of −0.042, equaled a point estimate of €38 433.81. While the ICUR point estimate was located in the lower-left quadrant of the cost-effectiveness plane (eFigure 2 in Supplement 1), the ICUR variance spread over all 4 quadrants, with a maximum of 72% in the lower-left quadrant, 22% in the upper-left quadrant, 1% in the upper-right quadrant, and 4% in the lower-right quadrant of the cost-effectiveness plane.

The cost-effectiveness acceptability curve is shown in eFigure 3 in Supplement 1. The curve indicated that IHT compared with inpatient treatment would be accepted as cost-effective at an MWTP threshold of €25 000 with a probability of 60%, and at an MWTP threshold of €50 000 with a probability of 44%.

The high level of stochastic uncertainty indicated by the ICUR variance and the low acceptability rates resulted in substantial value of additional information parameters revealed by the value of information curve (eFigure 4 in Supplement 1). The VOIP amounted to €688 at a MWTP threshold of €25 000 and €885 at a MWTP threshold of €50 000.

## Discussion

To our knowledge, this economic evaluation is the first CUA from the perspective of statutory health insurance and the societal perspective comparing IHT with inpatient treatment of people with mental illness in acute crisis. Consistent with health economic evaluations of other community-based acute psychiatric treatment approaches,^34,35,36,37,38^ we found that IHT was associated with a shift of psychiatric treatment costs from inpatient to outpatient care.^11^ However, in accordance with a previous study on the cost-effectiveness of an IHT program in the Netherlands,^20^ we found no significant differences in total societal costs or in costs covered by statutory health insurance between our study groups. These results indicate that acute psychiatric crisis treatment is very resource intensive, regardless of whether it takes place in a clinical setting or at the patient’s home.

The ICURs from the perspective of statutory health insurance and the societal perspective were both located in the lower-left quadrant of the cost-effectiveness plane, indicating that a loss of 1 QALY was associated with lower costs. Since the differences in QALY losses and the cost differences were not significant, the ICUR for both perspectives must be interpreted in consideration of the stochastic uncertainty. For this purpose, we estimated the cost-effectiveness acceptability curve. The results showed the probability that IHT in comparison with inpatient treatment for the treatment of persons with severe mental illness in acute crisis would be accepted as cost-effective at the MWTP threshold range between €25 000 and €50 000 that was recently suggested by Pichon-Riviere et al^33^ for the German health care system.

As indicated by the cost-effectiveness acceptability curve from the statutory health insurance perspective, we found a probability of 67% that for patients eligible for IHT in acute psychiatric crises, IHT would be cost-effective in comparison to inpatient treatment if the health insurance is willing to pay €25 000 for the gain of 1 life-year in full health. From the societal perspective, the acceptability rate would be 60% at an MWTP of €25 000 and 44% at an MWTP of €50 000. These acceptability rates are similar to those reported by Barakat and colleagues^20^ for the Dutch health care system. Results of our VOIP indicated that investments in further studies may be valuable. Given that the number of people with severe mental illness in Germany is estimated at 1% of the population,^39^ even with an eligibility rate of 50%, IHT could be a treatment option for about 500 000 patients. That would amount to a value of perfect information of €344 million if there was an MWTP of €25 000 for the gain of 1 QALY.

In contrast to the Netherlands, the UK, and other Organisation for Economic Co-operation and Development countries, WTP thresholds for QALY gains are not accepted by health policy decision makers as the basis of health care resource allocation in Germany.^40^ On the other hand, the efficiency-frontier approach preferred in Germany^41,42,43^ has been mainly developed to determine adequate reimbursement rates for new drugs but has been criticized for not providing a clear basis for the monetary valuation of health care outcomes^44^ and is therefore not used in many other countries. Nevertheless, our results provide health care decision makers an opportunity to consider the health economic outcome of IHT in an international context.

### Strengths and Limitations

To our knowledge, we conducted the first CUA of the German version of IHT for patients with acute psychiatric crises from the perspective of statutory health insurance and the societal perspective. We included a sufficient sample size and estimated total societal costs and QALYs across 12 months.

This study has limitations, including those resulting from the nonrandomized assignment of study participants and from not blinding participants and study workers to participant treatment assignments. Randomization of participants in acute psychiatric crisis is regarded as unethical. In addition, as part of the benefits package of statutory health insurance IHT in Germany, patients who opt for IHT cannot be randomized into a study group with immediate inpatient admission. Blinding to study treatment was not useful because of the obvious nature of the respective treatments. We applied propensity score matching to reduce selection bias by estimating the probability of receiving IHT compared with inpatient treatment conditional on the number of days previously spent in a psychiatric hospital or IHT, main psychiatric diagnosis, age, and gender. Both study groups were therefore comparable with regard to important determinants of potential selection bias. However, even using this procedure, we cannot exclude selection bias due to other nonmeasured characteristics.

## Conclusions

This economic evaluation found a small probability that IHT for persons with severe mental illness was cost-effective compared with inpatient treatment from the perspective of the statutory health insurance. However, due to the high stochastic uncertainty, the study results suggest that more research is needed to more clearly assess the economic efficiency of IHT.

## Notes

Supplement 1. **eTable 1.** Unit Cost List for the German Health Care System- 23.11.2022 **eTable 2.** Mixed Effects Regression Model for the EQ5D Index Values From Baseline to 12 Month Follow-Up **eFigure 1.** EQ5D Index Values (Means and 95 % CI) From Baseline to 12-Month Follow-Up **eFigure 2.** Variance of the Incremental Cost Utility Ratio (ICUR) for the Cost-Effectiveness of Intensive of Implementing Home-Treatment (IHT) vs. Inpatient Treatment (IT) for People With Severe Mental Illness in Acute Crisis in the German Health Care System From the Societal Perspective **eFigure 3.** Cost-Effectiveness Acceptability Curve (CEAC) for the Cost-Effectiveness of Intensive Home-Treatment (IHT) vs. Inpatient Treatment (IT) for People With Severe Mental Illness in Acute Crisis in the German Health Care System From the Societal Perspective **eFigure 4.** Value of Information (VOI) Curve for the Cost-Effectiveness of Implementing Intensive Home-Treatment (IHT) vs. Inpatient Treatment (IT) for People With Severe Mental Illness in Acute Crisis in the German Health Care System

Supplement 2.
Data Sharing Statement
