# Supplementary material for: Cost Utility of Intensive Home Treatment Compared With Acute Psychiatric Inpatient Admission
Source: JAMA Netw Open. 2025 May 27;8(5):e2512465. doi: 10.1001/jamanetworkopen.2025.12465 (PMC12117458; doi:10.1001/jamanetworkopen.2025.12465)
Supplement: Supplement 1. — eTable 1. Unit Cost List for the German Health Care System- 23.11.2022 eTable 2. Mixed Effects Regression Model for the EQ5D Index Values From Baseline to 12 Month Follow-Up eFigure 1. EQ5D Index Values (Means and 95 % CI) From Baseline to 12-Month Follow-Up eFigure 2. Variance of the Incremental Cost Utility Ratio (ICUR) for the Cost-Effectiveness of Intensive of Implementing Home-Treatment (IHT) vs. Inpatient Treatment (IT) for People With Severe Mental Illness in Acute Crisis in the German Health Care System From the Societal Perspective eFigure 3. Cost-Effectiveness Acceptability Curve (CEAC) for the Cost-Effectiveness of Intensive Home-Treatment (IHT) vs. Inpatient Treatment (IT) for People With Severe Mental Illness in Acute Crisis in the German Health Care System From the Societal Perspective eFigure 4. Value of Information (VOI) Curve for the Cost-Effectiveness of Implementing Intensive Home-Treatment (IHT) vs. Inpatient Treatment (IT) for People With Severe Mental Illness in Acute Crisis in the German Health Care System [file jamanetwopen-e2512465-s001.pdf]

Supplementary Online Content

Waldmann T, Bechdolf A, Nikolaidis K et al. Cost utility of intensive home treatment compared with acute psychiatric inpatient admission. *JAMA Netw Open*. 2025;8(5):e2512465. doi:10.1001/jamanetworkopen.2025.12465

eTable 1. Unit cost list for the German Health care system- 23.11.2022

eReferences.

eTable 2. Mixed effects regression model for the EQ5D index values from baseline to 12 month follow-up

eFigure 1. EQ5D Index values (means and 95 % CI) from baseline to 12-month follow-up

eFigure 2. Variance of the incremental cost utility ratio (ICUR) for the cost-effectiveness of intensive of implementing home-treatment (IHT) vs. inpatient treatment (IT) for people with severe mental illness in acute crisis in the German health care system from the societal perspective

eFigure 3. Cost-effectiveness acceptability curve (CEAC) for the cost-effectiveness of intensive of implementing home-treatment (IHT) vs. inpatient treatment (IT) for people with severe mental illness in acute crisis in the German health care system from the societal perspective

eFigure 4. Value of information (VOI) curve for the cost-effectiveness of implementing intensive home-treatment (IHT) vs. inpatient treatment (IT) for people with severe mental illness in acute crisis in the German health care system

This supplementary material has been provided by the authors to give readers additional information about their work.

© 2025 Waldmann T et al. *JAMA Network Open*.

eTable 1. Unit cost list for the German Health care system- 23.11.2022 (to be continued)

Category	Details	Unit	Unit Costs (in €)	Source*	Year
Inpatient	Psychiatric hospital	1 day	250.00	GKV Spitzenverband[1]	2017
	Psychosomatic hospital	1 day	250.00	GKV Spitzenverband[1]	2017
	Somatic hospital	1 day	643.15	Federal office of statistics[2,3]	2017
	Psychiatric rehabilitation	1 day	250.00	GKV Spitzenverband[1]	2017
Day hospital	Psychiatric day-care	1 day	190.00	GKV Spitzenverband[1]	2017
	Psychosomatic day-care	1 day	190.00	GKV Spitzenverband[1]	2017
	Somatic day-care	1 day	190.00	GKV Spitzenverband[1]	2017
Stäb		1 day	95.89	BKG Anlage 1a zur Vereinbarung gem.§§ 113, 118 und 120 SGB V zu PIA vom 01.01.2022	2022
Outpatient services	Psychiatrist	1 visit (10 min)	21.63	KBV[4] – Seite 80 in Arztgruppe-EBM 2022, Quartal 2,	2022
	Psychotherapist and Psychologist	1 visit (first 10 min)	8.90	KBV – Seite 477 in EBM 2022, Quartal 1	2022
	Psychotherapist and Psychologist	Every additonal 10 min	17.35	KBV – Seite 479 in EBM 2022, Quartal 1	2022
	Psychotherapist and Psychologist	1h	95.65	KBV – Seite 479 in EBM 2022, Quartal 1 (8.90+(5+17.35)	2022
	General practitioner	1 visit	12.85	KBV[4]	2017
	Other practitioner	1 visit	19.16	KBV[4]	2017
	Forensic aftercare	1 visit	150.00	Official gazette of the ministry of justice Rhineland-Palatinate	2019
Complementary services	Occupational therapist	1 hour	73.41	GKV Spitzenverband (www.gkv-heilmittel.de) , Anlage 2, S.3)	2022
	Physiotherapist	1 hour	36,64	KBV[4]	2017
	Neurologist	1 hour	144,06	KBV[4]	2017
	Osteopath	40 min	105.00	Osteopaths association[5]	2017
	Homeopath	40 min	60.00	NAV-Virchow-association[6]	2013
	Alternative practitioner	30 min	12.30	Association of alternative practitioners[7]	2002
	Speech therapist	45 min	45,18	KVBB[8]	2017
	Endocrinologist	1 visit	23,69	KBV[4]	2017
	Diabetologist	1 visit	23,69	KBV[4]	2017

© 2025 Waldmann T et al. *JAMA Network Open*.

e Table 1 continued: Unit cost list for the German Health care system- 23.11.2022

	Art therapy	90 min	100.00	Holzmann[9]	2018
	Music therapy	50 min	40.00	Siebels[10]	2018
	Nutrition counselling	1 hour	80.00	Conze[11]	2018
	Socio-pedagogical family assistance	1 hour	20.18	See Social Worker	2022
	Counselling (from church or community services, for child guidance, couples, sexual abuse, drug abuse…)	1 visit	43.90	Regional office of Caritas Ulm - Personal communication	2017
	Self-help group	--	--	Not charged	
	Socio-psychiatric counselling (incl. PIA)	1 hour	95.89	BKG Anlage 1a zur Vereinbarung gem.§§ 113, 118 und 120 SGB V zu PIA vom 01.01.2022	2022
	Parent-child counselling center	1 hour	20.18	See Social Worker	2022
	Sheltered workshop	1 day	47.26	Sheltered Workshop Provider Günzburg – Personal communication	2018
	Assisted living	1 month	630.43	Association of rehabilitation Ulm	2017
	Painting therapy	1 hour	85.00	Hik Kunsttherapie	2021
	Home care nursing service	Each 15 min	11.34	www.pflegemobil.info	2020
	Social worker	60 min	20.18	https://oeffentlicher-dienst.info/tvoed/sue/	2022
	Sports- or Dancetherapy	1 hour	61.64	Grupp et al.	2015
	Legal guardian	1 hour	34.83	VBVG (Bundesministerium der Justiz und fu¨ r Verbraucherschutz. Gesetz über die Vergütung von Vormündern und Betreuern: VBVG [Law of the remuneration of legal guardians]. 2005.	2005
	Psychiatric assessment		80.00	"Justizvergütungs- und -entschädigungsgesetz vom 5. Mai 2004 (BGBl. I S. 718, 776), das zuletzt durch Artikel 17 des Gesetzes vom 25. Juni 2021 (BGBl. I S. 2154) geändert worden ist" https://www.gesetze-im-internet.de/jveg/BJNR077600004.html	2004/ 2021
	Night in police cell		282.47	"Besondere Gebührenverordnung BMI vom 2. September 2019 (BGBl. I S. 1359), die zuletzt durch Artikel 1 der Verordnung vom 10. September 2021 (BGBl. I S. 4429) geändert worden ist" https://www.gesetze-im-internet.de/bmibgebv/BJNR135900019.html	2019

© 2025 Waldmann T et al. *JAMA Network Open*.

e Table 1 continued: Unit cost list for the German Health care system- 23.11.2022					
	Religious practicioner (priest, imam, rabbi, etc.)	1 hour	26.80	Bei einer 41h- Studenwoche www.steuerklassen.com/gehalt/pfarrer	2021
	Group therapy	50 min/ 1 hour	19.64	https://www.kbv.de/html/1150_53605.php	2021

eReferences.

1. GKV Spitzenverband & DKG Berlin. Vereinbarung zum pauschalierenden Entgeltsystem für psychiatrische und psychosomatische Einrichtungen für das Jahr 2017 2017.

2. Statistisches Bundesamt (Destatis). *Durchschnittliche Verweildauer Krankenhaus* Available at: https://www.destatis.de/DE/ZahlenFakten/GesellschaftStaat/Gesundheit/Glossar/Verweildauer.html [accessed 24.01.2019].

3. Statistisches Bundesamt (Destatis). *Kosten der Krankenhäuser nach Bundesländern* Available at: https://www.destatis.de/DE/ZahlenFakten/GesellschaftStaat/Gesundheit/Krankenhaeuser/Tabellen/KostenKrankenhaeuserBL.html [accessed 24.01.2019].

4. KBV. *Einheitlicher Bewertungsmaßstab 2017* 2017.

5. Verband der Osteopathen Deutschland e.V. *Behandlung | Verband der Osteopathen Deutschland e.V. (VOD e.V.)* Available at: https://www.osteopathie.de/osteopathie-behandlung [accessed 06.06.2018].

6. NAV-Virchow-Bund - Verband der niedergelassenen Ärzte Deutschlands e.V. Homöopathie als Kassenleistung *der niedergelassene arzt* 2013**; 4**.

7. Fachverband Deutscher Heilpraktiker. Gebührenverzeichnis für Heilpraktiker (GebüH) 2002.

8. KVBB. *Preisliste für Leistungen der Logopädie* 2018.

9. Holzmann S. *Kunsttherapie München: Kostenübersicht* Available at: https://www.kunsttherapie-holzmann.de/kosten_preise.php; 2018 [accessed 12.06.2018].

10. Siebels S. *Preisliste - Musiktherapie* Available at: http://klangstudio.net/preisliste.html; 2018 [accessed 06.06.2018].

11. Conze C. *Kosten - Ernährungsberatung Dr. Charlotte Conze* Available at: http://www.conze-ernaehrung.de/Ernaehrungsberatung_5.html; 2018 [accessed 06.06.2018].

12. Kreisverwaltung Pinneberg. Kosten Sozialpädagogische Familienhilfe 2010.

13. BKG - Bayerische Krankenhausgesellschaft e.V. *Vereinbarung Psychiatrische Institutsambulanzen - Vergütung* Available at: https://www.bkg-online.de/psychiatrische-institutsambulanzen-in-bayern-pia; 2018 [accessed 18.12.2018].

© 2025 Waldmann T et al. *JAMA Network Open*.

eTable 2. Mixed effects regression model for the EQ5D index values from baseline to 12 month follow-up

```
Mixed-effects regression                            Number of obs     =      1,083
Group variable: id                                  Number of groups  =        400
                                                    Obs per group:
                                                                  min =          1
                                                                  avg =        2.7
                                                                  max =          3
                                                    Wald chi2(5)      =      18.49
Log pseudolikelihood =  -24.74589                   Prob > chi2       =     0.0024

                                (Std. err. adjusted for 10 clusters in siteitt)
--------------------------------------------------------------------------------
              |               Robust
      EQindex | Coefficient  std. err.      z    P>|z|     [95% conf. interval]
--------------+-----------------------------------------------------------------
          iht |
          IHT |  -.0192572   .0320667    -0.60   0.548    -.0821068    .0435924
              |
         time |
      6-month |   .0809977   .0275745     2.94   0.003     .0269528    .1350427
     12-month |   .0796001   .0388137     2.05   0.040     .0035266    .1556736
              |
     iht#time |
  IHT#6-month |  -.0369234   .0302222    -1.22   0.222    -.0961578    .0223109
 IHT#12-month |  -.0105244   .0339373    -0.31   0.756    -.0770403    .0559915
              |
        _cons |    .639414   .0281366    22.73   0.000     .5842672    .6945607
--------------------------------------------------------------------------------

------------------------------------------------------------------------------
                             |               Robust
  Random-effects parameters  |   Estimate   std. err.     [95% conf. interval]
-----------------------------+------------------------------------------------
id: Identity                 |
                  var(_cons) |   .0561909   .0081809      .0422414    .0747469
-----------------------------+------------------------------------------------
               var(Residual) |   .0326308   .0039768      .0256975    .0414348
------------------------------------------------------------------------------
```

© 2025 Waldmann T et al. *JAMA Network Open*.

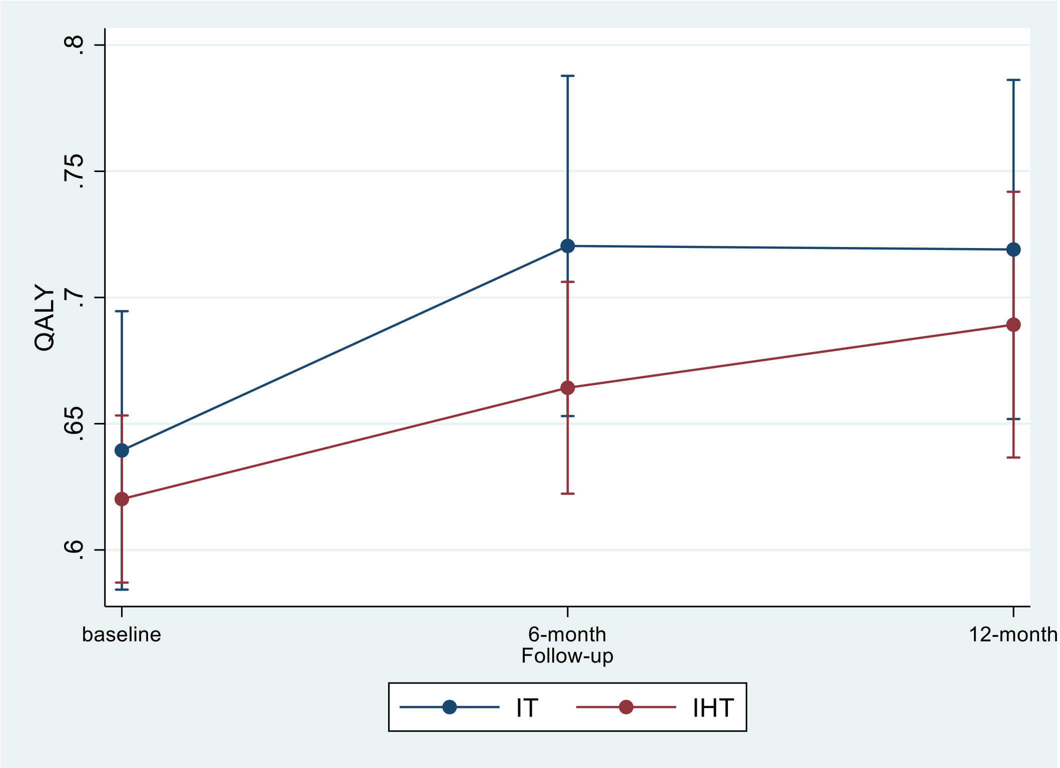

eFigure 1. EQ5D Index values (means and 95 % CI) from baseline to 12-month follow-up

© 2025 Waldmann T et al. *JAMA Network Open*.

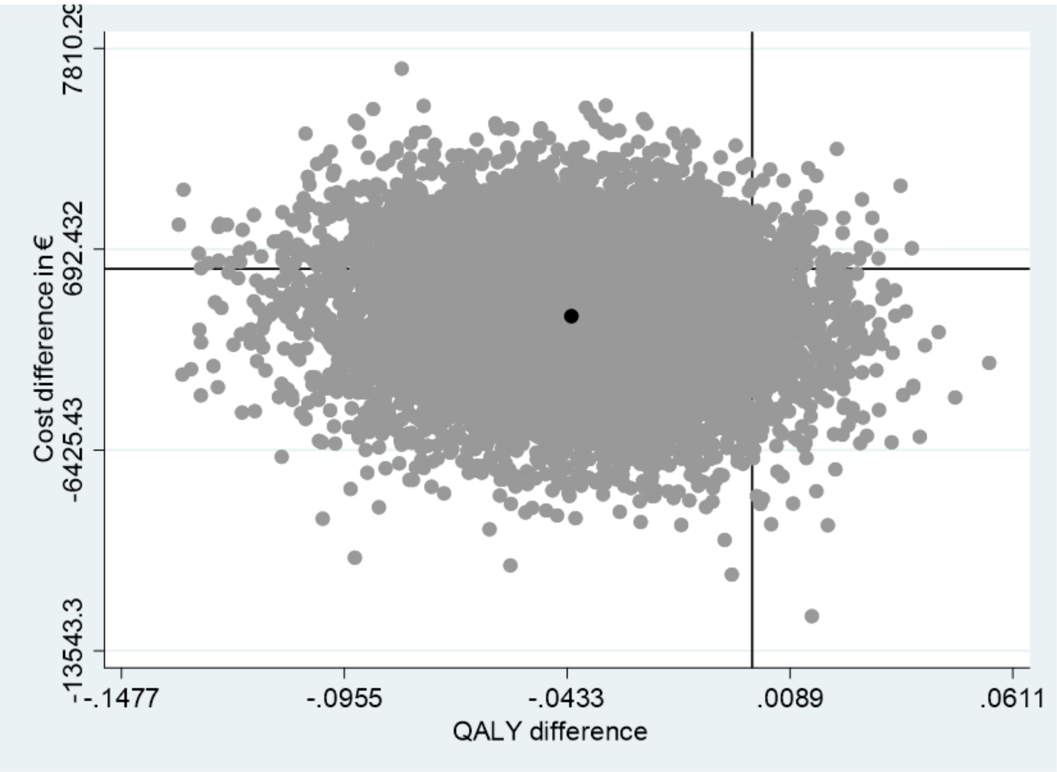

eFigure 2. Variance of the incremental cost utility ratio (ICUR) for the cost-effectiveness of intensive of implementing home-treatment (IHT) vs. inpatient treatment (IT) for people with severe mental illness in acute crisis in the German health care system from the societal perspective. The black dot represents the point estimate of the ICUR, each grey dot represents one of 10,000 ICURs estimated by non-parametric bootstrapping.

© 2025 Waldmann T et al. *JAMA Network Open*.

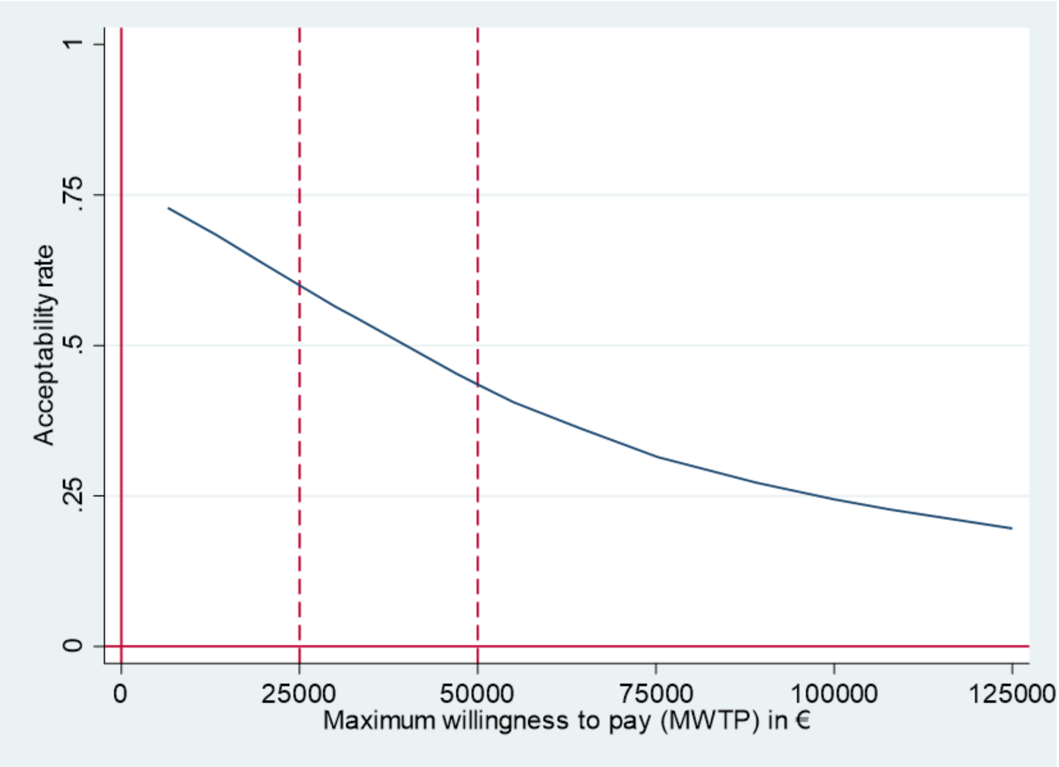

eFigure 3. Cost-effectiveness acceptability curve (CEAC) for the cost-effectiveness of intensive of implementing home-treatment (IHT) vs. inpatient treatment (IT) for people with severe mental illness in acute crisis in the German health care system from the societal perspective. The x axis represents the range of maximum willingness to pay (MWTP) threshold values. The dashed red vertical lines represent the MWTP thresholds of 25,000 € and 50,000 € suggested by for the German health care system. The y axis represents the acceptability rates, indicating the probability that IHT treatment would be accepted as cost-effective in comparison to IT in the German health care system. The acceptability curve represents the change of the acceptability rate with increasing MWTP threshold.

© 2025 Waldmann T et al. *JAMA Network Open*.

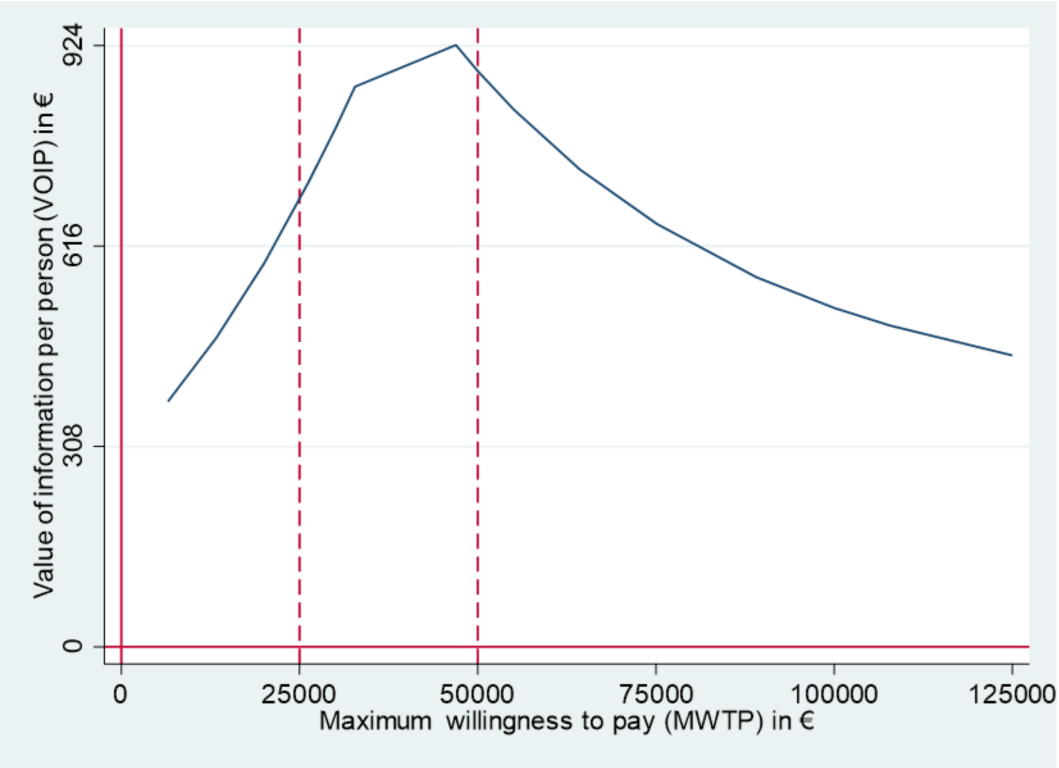

eFigure 4. Value of information (VOI) curve for the cost-effectiveness of implementing intensive home-treatment (IHT) vs. inpatient treatment (IT) for people with severe mental illness in acute crisis in the German health care system. The x axis represents the range of maximum willingness to pay in (MWTP) threshold values. The dashed red vertical lines represent the MWTP thresholds of 25,000 € and 50,000 € suggested by for the German health care system. The y axis represents the monetary value of acquiring perfect information per person (VOIP) in the population of potential service users. The curve represents the change of the VOIP with increasing MWTP thresholds.

© 2025 Waldmann T et al. *JAMA Network Open*.
