# Supplementary material for: Cost Utility of Intensive Home Treatment Compared With Acute Psychiatric Inpatient Admission
Source: JAMA Netw Open. 2025 May 27;8(5):e2512465. doi: 10.1001/jamanetworkopen.2025.12465 (PMC12117458; doi:10.1001/jamanetworkopen.2025.12465)
Supplement: Supplement 2. — Data Sharing Statement [file jamanetwopen-e2512465-s002.pdf]

# Data Sharing Statement

Waldmann. Cost-Utility of Intensive Home Treatment Compared With Acute Psychiatric Inpatient Admission. *JAMA Netw Open*. Published May 27, 2025.
doi:10.1001/jamanetworkopen.2025.12465

**Data**
**Data available:** Yes
**Data types:** Deidentified participant data, Data dictionary
**How to access data:** Request for data to corresponding author, Prof. Reinhold Kilian
**When available:** With publication

**Supporting Documents**
**Document types:** None

**Additional Information**
**Who can access the data:** Researchers whose proposed use of the data has been approved.
**Types of analyses:** for a specipied purpose
**Mechanisms of data availability:** with investigator support, after approval of a proposal
**Any additional restrictions:** none
